# Impaired Gal-9 Dysregulates the PBMC-Induced Th1/Th2 Imbalance in Abortion-Prone Matings

**DOI:** 10.1155/2018/9517842

**Published:** 2018-01-31

**Authors:** Mengzhou He, Ming Jiang, Yuan Zhou, Fanfan Li, Meitao Yang, Yao Fan, Yin Xie, Rajluxmee Beejadhursing, Ling Feng, Dongrui Deng

**Affiliations:** ^1^Department of Gynecology and Obstetrics, Tongji Hospital, Tongji Medical College, Huazhong University of Science and Technology, Wuhan, Hubei, China; ^2^Department of Reproductive Medical Center, Tangdu Hospital, The Fourth Military Medical University, Xi'an 710038, China

## Abstract

Recurrent miscarriage is defined as the loss of 3 or more consecutive pregnancies; however, the underlying immunologic mechanisms that trigger pregnancy loss remain largely unelucidated. Galectin-9 (Gal-9) may modulate a variety of biologic functions and play an important role in Th1/Th2 immune deviation. To analyze the mechanism of Gal-9 in abortion, we used the classical abortion-prone mouse model (DBA/2-mated CBA/J mice) to detect the expression of Gal-9 at the maternal-fetal interface. We also mimicked the immune environment of pregnancy by culturing trophoblast cells with peripheral blood mononuclear cells (PBMCs) to explore how Gal-9 might be involved in the pathogenesis of abortion. We found that the expression levels of Gal-9 in abortion-prone matings were lower than that for controls. Using a coculture system, we detected a Th1 preponderance in the coculture from abortion-prone matings. Furthermore, Gal-9 blockade augmented the imbalance of Th1/Th2 immunity in abortion-prone matings by promoting the secretion of Th1-derived cytokines in coculture, while there was a Th2 preponderance when we administered recombinant Gal-9. In conclusion, our results suggest that the Gal-9 signal is important for the regulation of PBMC function toward a Th2 bias at the maternal-fetal interface, which is beneficial for the maintenance of a normal pregnancy.

## 1. Introduction

Recurrent miscarriage, defined as the loss of 3 or more consecutive pregnancies, affects about 1–5% of couples [1]. The causes of abortion include genetic abnormalities, endocrine disorders, infection, and abnormal anatomy [2]. Although researchers increasingly theorize that a maternal immune imbalance correlates with miscarriage [3, 4], the underlying immunologic mechanisms that trigger pregnancy loss are largely unknown.

Maternal immune tolerance to semiallogeneic trophoblast cells of fetal origin is required for successful implantation and maintenance of pregnancy [5], and multiple mechanisms are believed to promote maternal-fetal immune tolerance. A number of studies have reported that defects in the balance of Th1/Th2 together with overfunctioning of Th1 are involved in abnormal immune responses and pathologic pregnancies, including miscarriage, preeclampsia, and fetal growth restriction [6]. The production of Th2-type cytokines, such as interleukin-4 (IL-4), IL-5, and IL-10, locally at the maternal-fetal interface assists in maintaining pregnancy; while Th1-type cytokines, such as interferon-*γ* (IFN-*γ*), IL-2, and tumor necrosis factor-*α* (TNF-*α*) mediate fetal rejection [7].

The placenta is an important component of the maternal-fetal interface. In mice, the placenta is composed of trophoblast giant cells (TGC), spongiotrophoblast cells (ST), and glycogen trophoblast cells [8]. To survive, these trophoblast cells would interact with the maternal immune system [9]. It is reported that peripheral blood mononuclear cells (PBMCs) can be recruited and induced to display unique phenotypic properties in the microenvironment at the maternal-fetal interface. By expressing multiple cell surface molecules, secreting a variety of soluble factors, and interacting with trophoblast cells, PBMCs participate in maintaining maternal-fetal tolerance, trophoblast invasion, and vascular remodeling [10, 11]. Abnormal changes in immune cell number and function are found to be closely related to adverse pregnancy outcomes, such as recurrent spontaneous abortion.

Galectin-9 (Gal-9), a member of the *β*-galactoside-binding lectin family, is expressed in the epithelium of the gastrointestinal tract, trophoblast cells, and several types of immune cells [12–14]. An increasing amount of evidence suggests that Gal-9 may modulate a variety of biologic functions and play an important role in immune response [15, 16]. For example, it has been shown that Gal-9 ameliorates glomerulonephritis or herpes simplex virus-induced inflammation by inhibiting Th1 and Th17 immune responses in mice [17, 18]. Gal-9 is reported to bind to a variety of molecules that exert a variety of physiologic and pathologic functions such as cell differentiation, apoptosis, and cytokine secretion [19]. In addition, it has been reported that rhGal-9 suppressed the production of Th1-type cytokine TNF-*α* and promoted the secretion of Th2-type cytokine IL-4 to control severe inflammatory responses induced by lipopolysaccharide [20]. It is suggested that Gal-9 may therefore participate in the regulation of Th1/Th2 balance.

Given the features of Gal-9, it may be an important regulator that plays a vital role in abortion by affecting the immunologic deviation in the Th1/Th2 ratio at the maternal-fetal interface. In order to establish a comprehensive study of immunity at both systemic and local levels, we herein wished to demonstrate the behavior of Gal-9 in the classical abortion-prone mouse model (DBA/2-mated CBA/J mice) from the perspective of immunology.

## 2. Materials and Methods

### 2.1. Mouse Models of Pregnancy

Female CBA/J mice and male DBA/2 mice (8–10 weeks old) were purchased from the Jackson Laboratory. Male BALB/c mice (8–10 weeks old) were obtained from Tongji Hospital (Wuhan, Hubei, China). All mice were kept under specific pathogen-free conditions. The care and use of experimental animals were conducted in accordance with the guidelines of the Chinese Council for Animal Care. Female CBA/J × male BALB/c matings (*n* = 10) were used as the normal pregnant model, and female CBA/J × male DBA/2 matings (*n* = 10) were used as the abortion-prone model that shares features of human spontaneous abortion [21, 22]. Pregnancy was determined by the presence of a vaginal plug, which was considered to be day 0.5 of pregnancy (E0.5). The mice were killed in 14.5 days of pregnancy, and embryo resorption rate was counted: *R* = *R*_e_/(*R*_e_ + *F*) (*R*_e_ is the number of embryo that was resorpted and *F* is the number of embryo that survived). Placentae and blood were obtained from female mice killed on E14.5. Portions of the placentae were preserved in 4% formaldehyde solution for immunochemistry, while the remainder was frozen in liquid nitrogen and used for Western immunoblotting and RT-PCR. Placentae served as primary cultures were conserved under sterile conditions.

### 2.2. Immunohistochemical Analysis

Immunohistochemical studies were performed on 5 *μ*m thick sections acquired from paraffin-embedded samples. The sections were baked, dewaxed, rehydrated, and incubated with 3% H_2_O_2_ to block endogenous peroxidase activity and then blocked with 1% bovine serum albumin (BSA) in phosphate buffer saline (PBS) to block endogenous peroxidase activity. The slides were incubated overnight at 4°C with primary antibodies: mouse anti-Gal-9 (1 : 50, Abcam Biosciences Incorporation, UK) for placentae and primary culture trophoblasts and mouse anti-CK-7 (1 : 30, Bios, China) for cellular identification. After washing, sections were incubated with HRP-labeled secondary antibodies at optimal concentrations for 30 min at 37°C. Negative controls were processed following substitution of the primary antibody with the corresponding concentrations of nonimmunized IgG. The reaction was developed with diaminobenzidine (DAB). Images were analyzed with Image-Pro Plus 6.0 (Media Cybernetics, Denver, USA), and the mean values for area-integrated optical densities were analyzed.

### 2.3. Western Immunoblot Analysis

Placental tissues were homogenized in RIPA buffer (Beyotime, China). Equal amounts of proteins were subjected to 10% SDS-PAGE and electrophoretically transferred to polyvinylidene difluoride membranes (Millipore Biosciences, America). After blocking with 5% skim milk for 1 h in Tris-buffered saline with 1% Tween 20 at room temperature, the blots were incubated overnight at 4°C with anti-Gal-9 (diluted 1 : 500 in 1% BSA in TBST). Membranes were then washed with TBST, followed by incubation with horseradish peroxidase-conjugated secondary antibodies (diluted 1 : 2000 in 1% BSA in TBST) for 60 min. *β*-Actin was used as a control for lane loading and was incubated with anti-*β*-actin primary antibody (diluted 1 : 1000 in 1% BSA in TBST) overnight and anti-mouse IgG peroxidase-conjugated secondary antibody (diluted 1 : 2000 in 1% BSA in TBST) for 60 min. Immunoblots were developed using Supersignal ECL (Amersham Biosciences, USA). Densitometric analyses were performed using Image Lab software (Bio-Rad Laboratories, USA).

### 2.4. Analysis of mRNA Expression by Real-Time PCR

The TRIzol regent (Invitrogen Life Technologies, USA) was used to extract RNA from placental tissues, and equal amounts of RNAs were reverse transcribed. The purity of the extracted RNA was tested photometrically, and the optical density (OD) at 260/280 nm was 1.8–2.0. The cDNAs were then amplified according to reverse kit (TOYOBO, Japan) instructions. A 20 *μ*L reaction contained 1 *μ*g template RNA (2 *μ*L), 1 *μ*L oligo(dT)_18_ primer (final concentration 5 pM), 9 *μ*L diethyl pyrocarbonate- (DEPC-) treated water, 4 *μ*L of 5x reaction buffer, 1 *μ*L RiboLock RNase inhibitor (20 U·*μ*L^−1^), 2 *μ*L of 10 mM dNTP mix, and 1 *μ*L RevertAid M-MuLV Reverse Transcriptase (200 U·mL^−1^). Reactions were incubated at 65°C for 5 min, 42°C for 60 min, and 70°C for 5 min, and then were instantly cooled on ice. The primers were designed with computer assistance according to GenBank sequences. The primers for Gal-9 were as follows: 5′-AGAGCGAAGTCTGCTTGGGAGG-3′ (forward) and 5′-GCAAGTTCTTCAGGCGGTGG-3′ (reverse). Each expression level was normalized to the housekeeping gene *β*-actin, using the primers 5′-CTGAGAGGGAAATCGTGCGT-3′ (forward) and 5′-CCACAGGATTCCATACCCAAGA-3′ (reverse). Real-time PCR was performed in a final volume of 20 *μ*L containing 10 *μ*L of 2x SYBR Premix Ex Tap (DBI, Nuremberg, Germany), 0.8 *μ*L of 2.5 mM primer, 2 *μ*L DNA template, and 7.2 *μ*L distilled H_2_O. The PCR was implemented according to the following parameters: 95°C for 3 min, 40 cycles at 95°C for 15 s, 60°C for 30 s, 72°C for 30 s, 60°C for 60 s, and 95°C for 15 s.

### 2.5. Isolation of Trophoblasts

The maternal-fetal units were harvested and removed from the uterine implantation sites on day E14.5. After removal of the decidua, the placental tissues were cut into pieces and enzymatically digested by compound enzyme (0.25% trypsin (Sigma, St. Louis, USA) and 50 U DNase I (Sigma, USA)) at 37°C for 20 min. The cell suspensions were loaded and separated on a discontinuous Percoll gradient (65–20%) and centrifuged at 800*g* for 25 min. The cellular fraction between 1.045 and 1.065 g/mL density was recovered and washed in DMEM-F12, which was supplemented with 10% fetal bovine serum (FBS) (Hyclone, USA) and 1% penicillin and streptomycin (Beyotime, Shanghai, China) [23]. Cells were resuspended in complete DMEM-F12 and maintained at 37°C in a humidified atmosphere of 5% CO_2_ in air. Six h after seeding, the medium was changed to remove any unattached cells and debris. Trophoblasts were assessed by immunocytochemistry for CK7 (trophoblast cell marker) positivity. We detected the expression of Gal-9 on primary trophoblasts from abortion and normal groups by flow cytometry. The cells were washed and incubated with PE IgG-control (R&D, USA) or PE anti-Gal-9 (BioLegend, USA) for 30 min at 4°C for flow cytometry. Parameters were obtained from a Calibur flow cytometer (BD Biosciences, USA) and analyzed by Cell Quest software (BD Biosciences, USA).

### 2.6. Isolation of PBMCs and Coculture Experiments with PBMCs and Trophoblasts

Blood of abortion and normal groups were acquired by retro-orbital vein puncture. After neutralization and filtration (38 *μ*m), the filtrate was laid carefully on Lymphocyte Separation Media (MD Pacific, Tianjin, China) and then centrifuged at 2000 rpm for 20 min; the PBMCs were collected from the interphase and washed twice with serum-free RPMI 1640 medium (Hyclone, Logan, Utah, USA) and centrifuged at 1000 rpm for 10 min. Cells were resuspended in complete RPMI 1640 medium. We collected PBMCs from abortion and normal groups to examine Gal-9 expression on PBMCs by flow cytometry.

Mouse primary trophoblasts from abortion and normal groups were cultured with their PBMCs, respectively, at a ratio of 1 : 1 (trophoblasts to PBMCs). We simultaneously collected the supernatants on days 1, 3, and 6. The Th1/Th2 cytokines were tested using ELISA on days 1, 3, and 6. The coculture system from abortion groups were subjected to anti-Gal-9 neutralizing antibodies (Abs) (5 *μ*g/mL, Abcam, Cambs., UK), Gal-9-Ig (10 *μ*g/mL, R&D Systems, Minneapolis, MN, USA), and control treatments. The Th1/Th2 cytokines were also tested on days 1, 3, and 6 using ELISA.

### 2.7. ELISA

The sera and coculture supernatants were collected and kept at −80°C until used. Cytokines from Th1/Th2 cells, such as IFN-*γ*, IL-2, and IL-4, were measured by ELISA kit (Dakewe, China) according to the manufacturer's instructions. Absorbance was detected using a microplate reader (Thermo Fisher, USA) at 450 nm.

### 2.8. Data Analysis

All data are presented as means ± SEM. Values were compared by Student's *t*-test for paired variables or by two-way ANOVA analysis to determine significance between/among respective groups with GraphPad Prism 5.0 (GraphPad Software Inc., La Jolla, CA). *p* < 0.05 was considered to be statistically significant.

## 3. Results

### 3.1. Low-Level Expression of Gal-9 in Placentae from Abortion-Prone Matings

**Figure 1:**
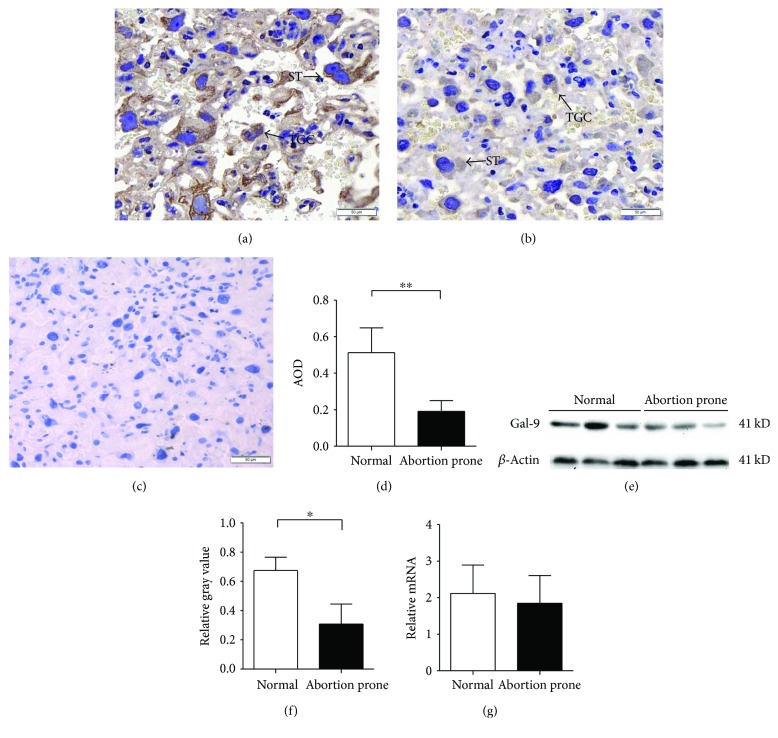
Expression of Gal-9 on placenta. Immunohistochemical localization of Gal-9 was shown on (a) normal (*n* = 10) and (b) abortion-prone (*n* = 10) matings, with (c) negative controls (400x) in placental tissues. The brown particles represent positive expression of Gal-9 protein. Gal-9 was distributed intracellularly and on cellular surfaces of trophoblast giant cells and spongiotrophoblast cells (arrows); and average optical density (AOD, AOD = integrated optical densities/area) between the two groups showed downregulated Gal-9 expression in abortion-prone matings (d). Placental tissues were examined by Western immunoblotting (e, f). The quantification of gray values between the two groups was in accordance with the immunohistochemistry (*n* = 15). (g) Real-time PCR indicated that there was no difference in the expression of Gal-9 in the placenta between the two groups (*n* = 10). ^∗∗^*p* < 0.01; ^∗^*p* < 0.05.

The abortion rate in CBA/J × DBA/2 matings and CBA/J × BALB/c matings were 22.4% (19/85) and 7.8% (8/102) in our study, respectively. The percentage of embryo resorption in CBA/J × DBA/2 matings was significantly higher than that in CBA/J × BALB/c matings (*p* < 0.05). It supports the hypothesis that these mice are prone to spontaneous abortion. Immunohistochemistry was performed primarily to examine the location of Gal-9 in placentae. Protein levels and mRNA expression of Gal-9 in placentae were compared in normal groups and abortion-prone groups by Western immunoblotting and RT-PCR. We found that clear and strong immunoreactivity for Gal-9 was observed in placental tissues, especially in trophoblast giant cells and spongiotrophoblast cells (Figures 1(a)–1(c)). We also observed using immunohistochemistry and Western blotting analysis that the CBA/J × DBA/2 matings showed significantly reduced Gal-9 signals in placentae compared with CBA/J × BALB/c matings (Figures 1(d)–1(f)), while there was no difference in mRNA expression between the abortion-prone group and normal pregnancies (Figure 1(g)).

### 3.2. Downregulated Expression of Gal-9 in Primary Isolated Trophoblasts in Abortion-Prone Matings

**Figure 2:**
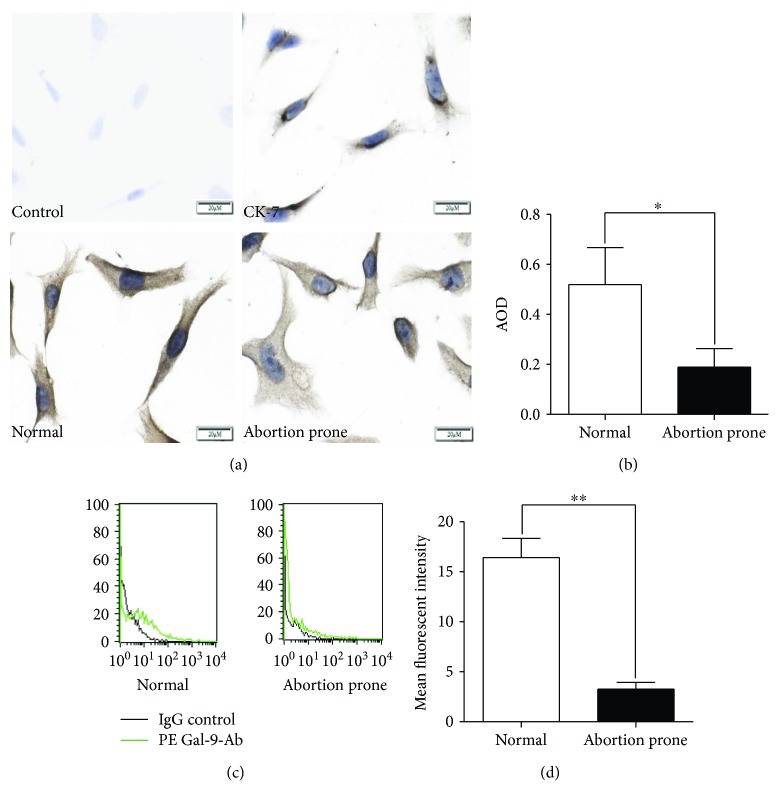
Gal-9 expression on primary cultured trophoblasts. (a) Expression of Gal-9 on primary cultured trophoblast cells was observed by immunohistochemistry (bar = 20 *μ*m) in normal pregnancy and abortion-prone matings. Trophoblastic cells were identified by CK-7. (b) AOD showed that Gal-9 was downregulated in abortion-prone groups in trophoblasts, and flow cytometry measurements demonstrated Gal-9 protein on trophoblast cell surfaces (c, d). There appeared to be an obvious decrease in mean fluorescence intensity of Gal-9 among trophoblasts from abortion-prone matings (*n* = 5). ^∗∗^*p* < 0.01; ^∗^*p* < 0.05.

Trophoblasts isolated from placentae form both groups were identified by CK-7, and the expression of Gal-9 in trophoblast cells was analyzed by immunochemistry (Figures 2(a) and 2(b)). We found that Gal-9 was detected in isolated trophoblasts, with the strongest labeling in the membrane and relatively lower labeling in the cytoplasm. There was a significantly lower expression of Gal-9 in abortion-prone group than in the normal groups. In order to determine whether Gal-9 exerted a discernible effect on trophoblast cell surfaces, freshly isolated trophoblasts were examined by flow cytometry and as expected, trophoblasts of mice from abortion-prone matings showed a considerably low-level expression of Gal-9 on trophoblasts (Figures 2(c) and 2(d)).

### 3.3. Th1 Preponderance in the Coculture of PBMCs and Trophoblasts from Abortion-Prone Matings

**Figure 3:**
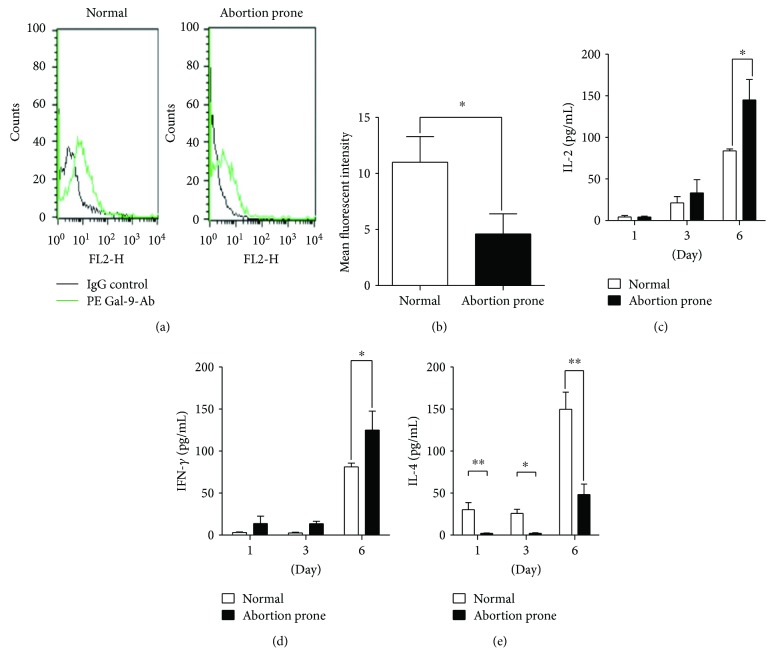
Th1 preponderance in the coculture system derived from mice of abortion-prone matings. Flow cytometry measurements of Gal-9 protein on PBMCs. We show intuitive images with the green line representing the addition of PE-Gal-9-Ab and the black line representing the addition of IgG-control (a). The mean fluorescence intensity showed a downregulated Gal-9 expression on PBMCs from abortion-prone matings (b). PBMCs were cocultured with trophoblasts. IL-2 (c), IFN-*γ* (d), and IL-4 (e) were evaluated in coculture by ELISA at d 1, d 3, and d 6. There was higher IL-2 and IFN-*γ* production and lower IL-4 production from abortion-prone matings than from controls at d 1, d 3, and d 6 (*n* = 5). ^∗∗^*p* < 0.01; ^∗^*p* < 0.05.

We tested the expression of Gal-9 on PBMCs cell surfaces to discern a relationship between Gal-9 and maternal immune response. Characterization by flow cytometry showed that PBMCs from normal groups exhibited a higher expression of Gal-9 than did the abortion-prone groups (Figures 3(a) and 3(b)).

To confirm the relationship between Gal-9 and Th1/Th2 in pregnancy, we cocultured PBMCs with trophoblasts and focused on cytokine production. We examined IL-2, IFN-*γ*, and IL-4 in the supernatants from the aforementioned coculture system. As expected, our observations showed that the levels of the Th1-type cytokines, IL-2 and IFN-*γ*, were elevated substantially, especially at 6 d, while the Th2-type cytokine, IL-4, was reduced relative to controls in cell coculture of mice from abortion-prone matings (Figures 3(c)–3(e)).

### 3.4. Effect of Gal-9-Targeted Intervention on the Cocultures of Normal versus Abortion-Prone Matings

**Figure 4:**
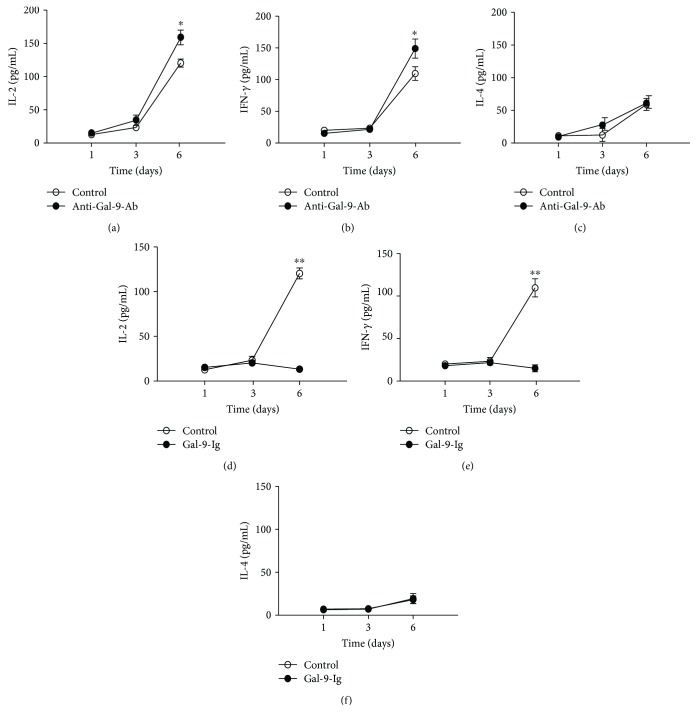
Effect of galectin-9 blockade and recombinant galectin-9 treatment on in vitro Th1/Th2 cytokine expression from coculture supernatants of abortion matings. IL-2 (a), IFN-*γ* (b), and IL-4 (c) expression in coculture supernatants of trophoblastic cells with PBMCs isolated from abortion-prone matings, with or without anti-galectin-9-Ab administration. IL-2 (d), IFN-*γ* (e), and IL-4 (f) expression in coculture containing trophoblastic cells and PBMCs, with or without recombinant Gal-9 administration. All cytokines were collected and detected at d 1, d 3, and d 6 by ELISA. All results are presented as means ± SEM of at least three independent experiments (*n* = 5). ^∗∗^*p* < 0.01; ^∗^*p* < 0.05.

We added anti-Gal-9-Ab or recombinant Gal-9-Ig to the coculture systems of abortion groups to further explore the potential effects of Gal-9 on the peripheral immune environment of pregnancy. Interestingly, in abortion-prone matings, we observed that Gal-9 blockade significantly increased production of IL-2 and IFN-*γ* levels compared with untreated groups (Figures 4(a) and 4(b)). However, IL-4 remained unchanged compared with untreated groups (Figure 4(c)). It is intriguing to note that recombinant Gal-9-Ig exerted an inhibitory effect on the production of Th1-type cytokines of abortion groups compared with untreated groups (Figures 4(d) and 4(e)). However, there was no difference in the secretion of IL-4 (Figure 4(f)).

## 4. Discussion

In the present study, we demonstrated that Gal-9 is essential for the maintenance of normal pregnancies via the regulation of the induction by PBMC of Th1/Th2 balance at the maternal-fetal interface. Gal-9 was found to be downregulated on placenta and PBMCs from miscarriages, and it displayed higher levels of Th1-type cytokines but lower levels of Th2-type cytokine production in PBMCs cocultured with their trophoblast cells, when taken from abortion-prone mouse matings. In addition, we also found that there were Th1-predominant responses in cocultures from abortion-prone mice, which are further enhanced by anti-Gal-9-Ab and abolished by Gal-9-Ig. Therefore, our results suggest that Gal-9 expression in trophoblasts and PBMCs is critical for normal pregnancy.

To obtain a successful pregnancy, trophoblasts must exhibit several functions, such as (i) providing a physical barrier [24], (ii) recruiting signals and suppressing maternal reactivity [25], (iii) producing immunosuppressive hormones locally [26], and (iv) enhancing the production of blocking factors that are able to bind to several antigenic sites [27]. Gal-9 thereby serves as an immune-tolerant subset. In our studies, we found that Gal-9 was widely expressed in trophoblast cells from mice and that there was a significantly enhanced Gal-9 expression by normal groups compared with abortion-prone groups. According to our hypothesis, the dominant presence of Gal-9 at trophoblast cells at the maternal-fetal interface suggests a subsequent local immunosuppressive potential following maternal immunoactivation.

It is widely believed that over half of women with recurrent abortion have increased Th1/Th2 cell ratios [28]. Thus, Th2 bias has been a common hypothesis used to explain pregnancy tolerance and has been regarded as an important marker of successful pregnancies. Many researchers reported that the Gal-9/Tim-3 pathway plays a vital role in the regulation of Th1/Th2 balance by inducing apoptosis of Th1 cells and subsequently suppressing the Th1-type immune response [29, 30]. Within the scope of our research, we discovered a notably increased Gal-9 expression in PBMCs from mice undergoing normal pregnancies relative to abortion-prone groups. In addition, there was greater IL-4 and reduced IL-2 and IFN-*γ* production in cocultures of trophoblast cells from normal pregnant mice. We speculate that Gal-9 might therefore be used as an important marker to indicate the tolerant state of PBMCs in the maternal milieu.

A currently supported hypothesis maintains that the ability of trophoblastic antigens to induce a natural and tolerogenic maternal response involves regulatory T cells, cytokines, chemokines, and galectin-1 derived from maternal-fetal tissue [8]. At the maternal-fetal interface, there exist many immune cells, including NK cells, T cells, and macrophages, and a majority of these cells are recruited from the periphery and are then “educated” by the maternal-fetal microenvironment. After “education,” the function of these cells focuses on providing support and protection for the developing fetus [1]. PBMCs are primarily responsible for fighting pathogen infections via the secretion of cytotoxic molecules [31], and after migration to the maternal-fetal interface, these cells participate in the maintenance of immune tolerance through the secretion of various types of cytokines [32]. Li et al. found that the coculture of peripheral blood NK cells (pNK cells) with their trophoblasts significantly increased Th2-type cytokine production and decreased Th1-type cytokine and perforin expression. This makes pNK cells more adaptable to an immune-tolerant phenotype [20]. In the present study, we cocultured PBMCs with their trophoblasts, and the PBMCs in the normal groups displayed an immune-tolerant trait characterized by the production of higher levels of IL-4 and lower IL-2 and IFN-*γ* than those in the abortion-prone group. We blocked Gal-9 with anti-Gal-9-Ab in the abortion-prone coculture groups and found that this led to a Th1 predominance. In addition, when we administrated Gal-9-Ig to our coculture system, there was a significantly decreased production of Th1-type cytokines, and this triggered a Th2 bias. Interestingly, IL-4 did not produce any visible change. We speculate that the stable secretion of IL-4 might be due to the excessive inflammatory response in abortion condition or the intricate relationship between Tim-3-Gal-9 and IL-4 which should be probed for deeper investigation. Consequently, we propose that Gal-9 may be responsible for maintaining this Th2-bias in PBMCs and promote fetal tolerance.

A large body of evidence suggests that spontaneous abortion is associated with a disturbed Th1/Th2 cytokine profile and defective immune cell function [7]. Collectively, our study demonstrated that Gal-9 signaling is critical to PBMC function in immune tolerance. The dysregulation of Gal-9 signaling in PBMCs and trophoblasts may be the main cause of miscarriages, indicating that alterations in the normal function of Gal-9 might be involved in the pathogenesis of these diseases. Therefore, Gal-9 might be a novel target of immunotherapy for pregnancy complications.
